# Tropical Storm Debby: Soundscape and fish sound production in Tampa Bay and the Gulf of Mexico

**DOI:** 10.1371/journal.pone.0254614

**Published:** 2021-07-13

**Authors:** Anjali D. Boyd, Shannon Gowans, David A. Mann, Peter Simard

**Affiliations:** 1 Eckerd College, Marine Science Discipline, St. Petersburg, Florida, United States of America; 2 Eckerd College, Marine Science and Biology Disciplines, St. Petersburg, Florida, United States of America; 3 Loggerhead Instruments, Sarasota, Florida, United States of America; 4 Eckerd College, Environmental Studies Discipline, St. Petersburg, Florida, United States of America; University of Windsor, CANADA

## Abstract

Tropical cyclones have large effects on marine ecosystems through direct (e.g., storm surge) and indirect (e.g., nutrient runoff) effects. Given their intensity, understanding their effects on the marine environment is an important goal for conservation and resource management. In June 2012, Tropical Storm Debby impacted coastal Florida including Tampa Bay. Acoustic recorders were deployed prior to the storm at a shallow water location inside Tampa Bay and a deeper water location in the Gulf of Mexico. Ambient noise levels were significantly higher during the storm, and the highest increases were observed at lower frequencies (≤ 500 Hz). Although the storm did not directly hit the area, mean ambient noise levels were as high as 13.5 dB RMS above levels in non-storm conditions. At both the shallow water and the deep water station, the rate of fish calls showed a variety of patterns over the study period, with some rates decreasing during the storm and others showing no apparent reaction. The rates of fish calls were frequently correlated with storm conditions (storm surge, water temperature), but also with lunar cycle. Reactions to the storm were generally stronger in the inshore station, although fish sounds increased quickly after the storm’s passage. Although this was not a major tropical cyclone nor a direct hit on the area, the storm did appear to elicit a behavioral response from the fish community, and ambient noise levels likely limited the abilities of marine species to use sound for activities such as communication. Given the increases in intensity and rainfall predicted for tropical cyclones due to climate change, further studies of the ecological effects of tropical cyclones are needed.

## Introduction

Tropical cyclones (e.g., tropical storms, hurricanes) can have ruinous effects on coastal ecosystems, including flooding and enhanced erosion, as well as detrimental effects on benthic and pelagic marine communities. The biological effects of tropical cyclones have been documented in a variety of taxa and habitats, including benthic communities (especially coral reefs [1] and fish [2]). However, the effects of tropical cyclones on marine communities are not well understood and appear to be dependent on a variety of factors including the intensity of the storm and the specifics of the habitat (e.g., coral reef habitat vs. seagrass beds), the timescale considered (e.g., whether observations were conducted days vs. months after the storm) and the methodology (e.g., visual surveys vs. acoustic monitoring).

Many studies have examined the effects of tropical cyclones on fish using traditional survey methods such as net sampling or visual counts. While traditional visual based survey techniques have advantages, they are difficult to implement immediately before, during or after tropical cyclones. Therefore, passive methodologies–where data are collected remotely–can be a useful tool. For example, acoustic telemetry tags have been used to document the distribution changes in red snapper (*Lutjanus campechanus*) and several species of shark in response to tropical storms [3–5]. Passive acoustic monitoring (PAM) devices can operate for long periods of time and in environmental conditions that make many other techniques difficult or impossible, such as during major storms [6]. For example, PAM technology was used to monitor fish sounds in Charlotte Harbor, Florida during Hurricane Charley, a category 4 hurricane [7]. The authors found that the hurricane did not inhibit fish sound production, despite the fact that the recorder was located in only 3.5 m of water.

An additional advantage of PAM is the ability to conduct soundscape analysis. Soundscape analysis is the characterization and quantification of natural and anthropogenic sounds in a given environment [8]. The study of soundscapes can involve both spatial and temporal analysis of sounds to assess natural non-biological conditions (the “geophony”: e.g., wind-driven waves, earthquakes), biological conditions (the “biophony”: e.g., species-specific sounds, biodiversity), and anthropogenic noise (the “anthrophony”: e.g., boat noise, marine construction; [8]). Furthermore, soundscapes can provide insight into the structure of biological communities, and how natural and anthropogenic disturbances effect community structure [8, 9].

In 2012, Tropical Storm Debby formed in the Gulf of Mexico and impacted Tampa Bay, Florida, from June 24^th^ -27^th^ [10]. In the Tampa Bay area, the storm was characterized by a storm surge, inundating rainfall, and sustained winds of over 54 km h^-1^. At the time of this storm, two bottom-mounted autonomous acoustic recorders were operating in the Tampa Bay area. As most previous studies investigating biological responses to tropical cyclones suffer from undersampling due to the inability to collect data at high-frequency intervals, especially during and immediately after the storm, PAM is an ideal methodology to examine the biological responses to and the recovery after these weather systems. As the frequency and severity of these storms is likely to increase with global climate change [11–13], our understanding of biological responses to these storms is increasingly important. Using the data from these acoustic recorders, the aims of this study were (a) to investigate the underwater soundscape associated with this tropical storm, and (b) to investigate changes in fish sound production over the duration of the storm. Based on the results from previous studies, we hypothesized that Tropical Storm Debby would cause an increase in ambient noise, and given the variability in the literature, we hypothesized a variety of species-specific patterns in fish vocalization rates.

## Materials and methods

### Sampling sites

**Fig 1:**
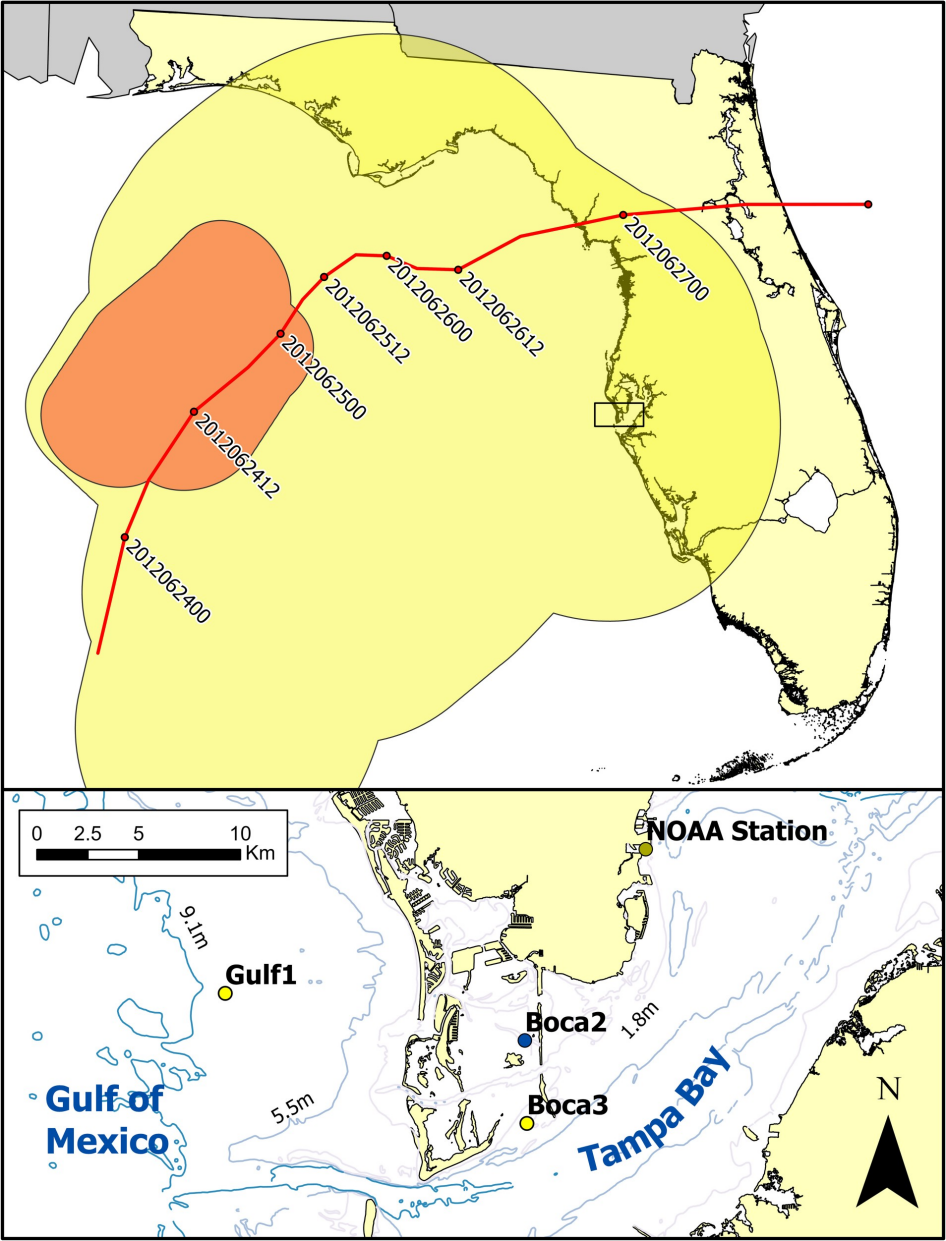
Tropical Storm Debby path. Top panel: track of the center of Hurricane Debby from June 24 (00:00) to June 27 (00:00) shown by red line (date-time coding of positions YYYYMMDDHH). Yellow polygon shows the NOAA working best track wind swath between June 23 and June 26 (≥34 knots). Red polygon shows the NOAA working best track wind swath between June 24 and June 25 (≥50 knots). Bottom panel: study area, showing the locations of the acoustic recorder stations, Boca 3 (inshore, ≈3 m depth) and Gulf 1 (offshore, ≈9 m depth), station Boca 2 (temperature data only, ≈3 m depth), and NOAA weather station St. Petersburg. Landform and bathymetry data from the Florida Fish and Wildlife Conservation Commission [14]; storm track and wind swath data from the NOAA National Hurricane Center / National Weather Service [15].

Acoustic recorders were deployed throughout the duration of Tropical Storm Debby at two sites: (1) station Boca 3, an inshore, shallow-water (approximately 3 m) sparse seagrass bed in Boca Ciega, Tampa Bay, and (2) station Gulf 1, an offshore, deeper water (approximately 9 m) sandy bottom site in the open Gulf of Mexico (approximately 10 km from shore, Fig 1). Both of these sites were approximately 175 km south of the storm track, although well within the ≥34 knot wind swath of the storm (Fig 1). Acoustic recorders were bottom-mounted, shackled to 1.2 m augers screwed into the sediment. These deployments were part of a longer-term study involving acoustic recorder deployments in a variety of locations in Tampa Bay and the Gulf of Mexico to monitor bottlenose dolphins (*Tursiops truncatus*) under National Marine Fisheries Service General Authorization for Scientific Research 1077–19540 & 1077–1794 and were not specifically deployed for the tropical storm. However, due both to the deployment areas and the species considered in this study (i.e., passively monitoring non-protected species in non-protected areas), no permits were required to deploy the recorders at these sites or to carry out our analysis.

The acoustic recorders were Digital SpectroGram [DSG] recorders (Loggerhead Instruments, Sarasota, FL, USA) with HTI-96-MIN hydrophones (-170 dB/V, High-Tech Inc., Long Beach, MS, USA). Recorders operated on a duty cycle of 10 seconds per 10 minutes, at a 50 kHz sample rate and 16-bit resolution, and data were stored on 32 GB SD cards.

In-situ water temperature data were collected using Hobo UA-002-64 temperature loggers (ONSET Corporation, Bourne, MA, USA). While a temperature logger was operational at the offshore station Gulf 1, one was not operational at the inshore station Boca 3. However, the nearby inshore station Boca 2 (approximately 4 km to the north, Fig 1) did have an operational temperature logger, although it did not have an operational DSG recorder during the time of the tropical storm. Therefore, temperature data from station Boca 2 was used in this study as a proxy for the temperature conditions at the acoustic recorder Boca 3. Temperature loggers recorded every 10 minutes and were located approximately 0.5 m above the sea floor.

### Additional environmental data

In addition to the in-situ water temperature data collected in this study, wind speed, water level, and barometric pressure were obtained from the US National Oceanic and Atmospheric Administration [16–18]. These data were recorded at the NOAA St. Petersburg Station 8726520, approximately 15 km to the north-northeast from Boca 3 (inshore) and approximately 20 km to the east-northeast from Gulf 1 (offshore). Moon phase data for the study period were obtained from the National Aeronautics and Space Administration [19] as a number of fish species have shown lunar periodicity in their call rates [20, 21].

### Analysis

In order to quantify the effects of the tropical storm during its passage through the study area, three time periods were defined: before, during and after the storm. Dates were selected based on the water level anomaly (storm surge), as this environmental variable indicated when tropical storm Debby had its greatest impact on our field sites. Water level anomaly was calculated by subtracting the observed water levels from the predicted water levels [16, 17]. Five days during the storm’s passage over the study area were characterized by having a water level anomaly above 0.5 m (June 22–26), while the five days before and after this period had water level anomalies below 0.5 m (June 17–21 and June 27-July 1, respectively).

Acoustic recordings from June 17th—July 1st were analyzed via (a) soundscape analysis using third-octave bands [22], and (b) spectrogram analysis of fish sounds [20]. Third-octave soundscape analysis was conducted in MATLAB 2009b (Mathworks, Natick MA, USA) and was used to characterize band-specific changes in ambient noise levels over the course of the storm. Individual sound files were band-pass filtered at standardized third-octave center frequencies established by the United Nations Economic Commission for Europe [23]. The mean root-mean-square (RMS) sound pressure level was calculated for each 24-hour period in each third-octave band, using the sensitivity of the recorder (0.1 V full-scale) and the hydrophone (-170 dBV re 1 μPa). Fish sounds were identified by manually inspecting 1024-point spectrograms in Raven Pro 1.5 (Cornell Lab of Ornithology, Ithaca, NY). The number of fish sounds were counted in each 10-second file, identified to species whenever possible. Published species-specific vocalization patterns from FishBase [24] and DOSITS’ Discovery of Sound in the Sea Audio Gallery [25] were used to identify fish species vocalization patterns.

Statistical analysis was conducted in RStudio (Version 1.3.1073, PBC). Repeated measures ANOVA tests with Tukey post-hoc comparisons were used to determine if differences in RMS sound pressure levels in individual files existed in each third-octave band before, during and after Tropical Storm Debby at both stations. Comparisons of the number of fish calls detected (total calls and species-specific calls) per individual file at each station between the three time periods (before, during and after the passage of Tropical Storm Debby) were also tested using repeated measures ANOVA tests with Tukey post-hoc comparisons. Spearman’s correlations were calculated between the total number of fish calls and species-specific calls for each 24-hour period of the study period at each station and the mean bottom temperature for each 24-hour period (collected at station Boca 2 for the inshore recorder), the mean sea level anomaly for each 24-hour period (i.e., storm surge, collected at the NOAA St. Petersburg weather station, [16, 17]) and the daily lunar cycle [19]. In addition, Spearman’s correlations were calculated between the total number of fish calls and species-specific calls for each 24-hour period and the mean RMS noise level at the 500 Hz third-octave band for each 24-hour period. This frequency band was chosen as it falls within the frequency band of most fish sound production (50–1000 Hz: e.g., [20, 26]), allowing us to better determine both if ambient noise levels were affecting fish sound production, and if acoustic masking by ambient noise was affecting our detection rates.

## Results

### Tropical Storm Debby conditions

**Fig 2:**
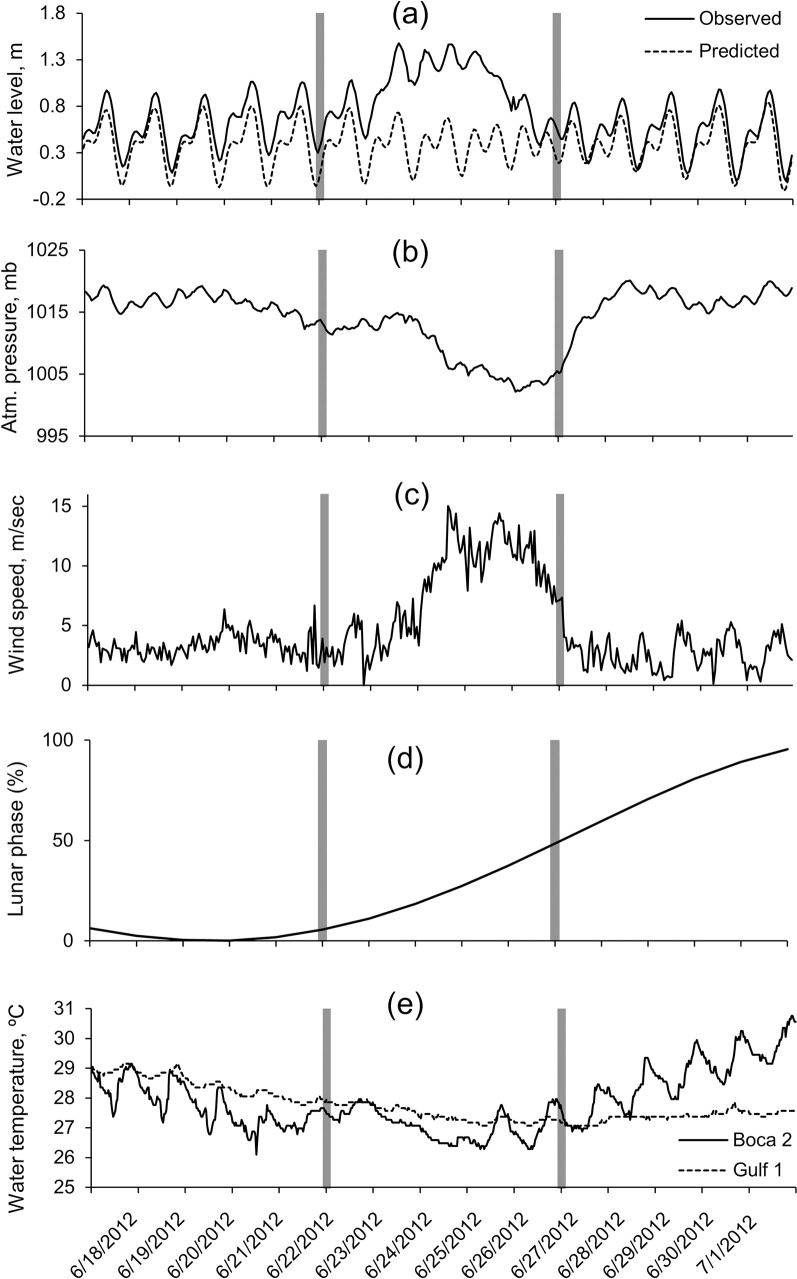
Tropical Storm Debby environmental conditions. Environmental conditions before, during and after the passage of Tropical Storm Debby (bars show the first day of the “during storm” period, and “after storm” period). (a) Observed and predicted water level (m), (b) atmospheric pressure (mbar), and (c) wind speed (m/sec) from the NOAA St. Petersburg station (data from [16–18]). (d) Percent of moon visible (data from [19]). (e) Temperature data collected at the acoustic recorder stations Boca 2 (inshore) and Gulf 1 (offshore).

At the NOAA St. Petersburg weather station, Tropical Storm Debby was characterized by a storm surge as high as 1.17 m above predicted levels ([16, 17] Fig 2A), a decrease in barometric pressure to 1002.2 mb ([18] Fig 2B), and wind speeds up to 15.0 m sec^-1^ ([19] Fig 2C). During this time period, the moon was waxing and increased from approximately 5% visible at the beginning of the study period to 93% visible at the end of the study period ([19] Fig 2D). At both stations Boca 2 (inshore) and Gulf 1 (offshore), water temperature generally decreased as the storm approached the study area (Fig 2E). At station Boca 2 the water temperatures ranged between 26.1°C and 30.8°C (range = 4.7°C). Water temperatures at this station had noticeable (approximately 2°C) diel fluctuations before and after the storm’s passage; however, these fluctuations were mostly absent during the storm. Water temperature increased rapidly after the passage of Tropical Storm Debby (increasing by approximately 4°C in five days). At station Gulf 1, the temperature range was less (27.0°C to 29.2°C, range = 2.2°C) and little diel temperature fluctuation was observed. The temperature increase after the passage of the storm at station Gulf 1 was minimal (Fig 2E).

### Soundscape analysis

**Fig 3:**
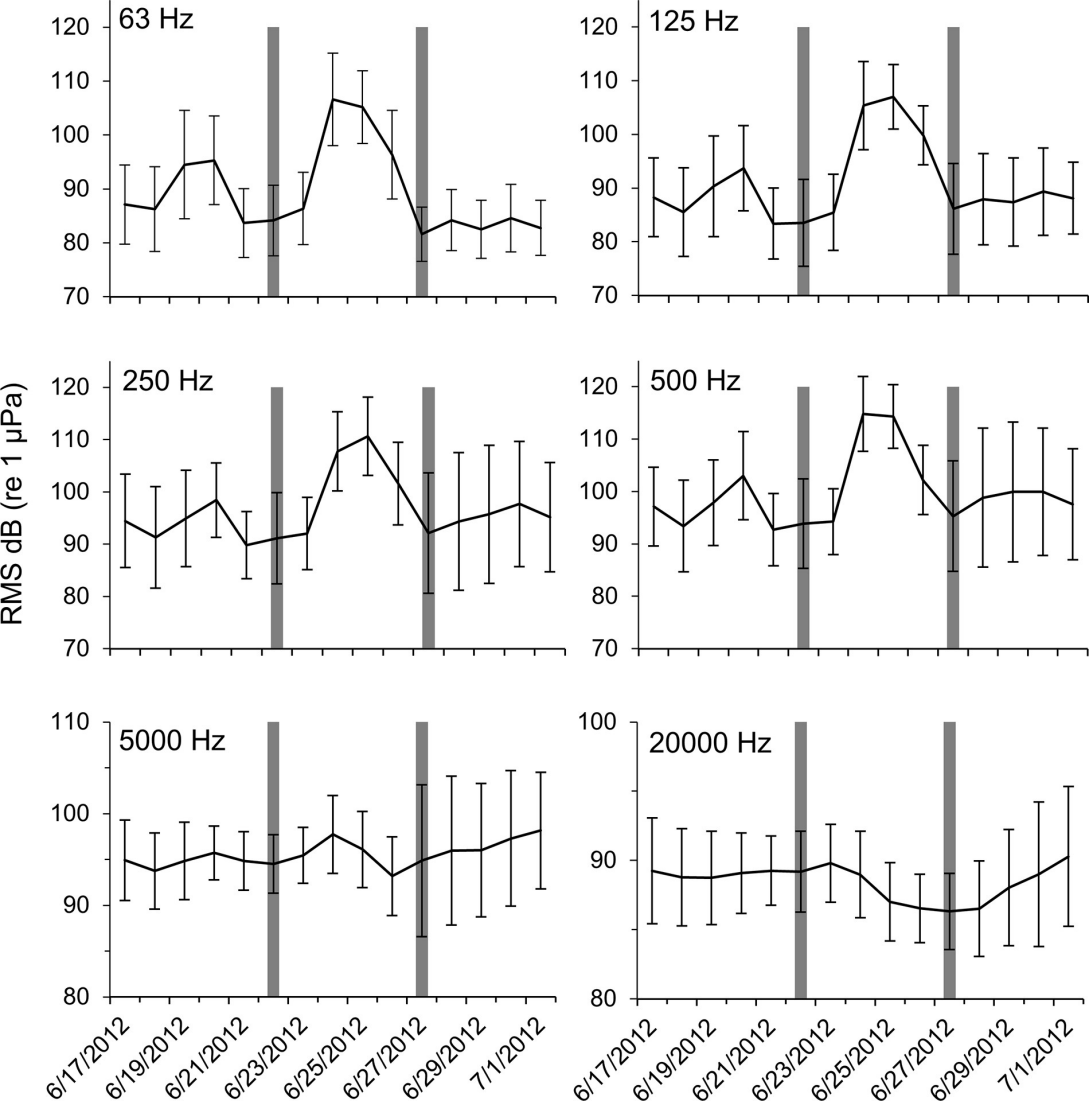
Boca 3 sound pressure levels during Tropical Storm Debby. Daily mean RMS sound pressure levels at station Boca 3 (inshore) for third-octave bands centered at 63 Hz, 125 Hz, 250 Hz, 500 Hz, 5000 Hz, and 20,000 Hz third-octave bands. Dark bars indicate the first day of the “during storm” period, and the first day of the “after storm” period. Error bars ± 1 SD. Note that the scale of the y-axes for the top four panels are not the same as the bottom two panels.

**Fig 4:**
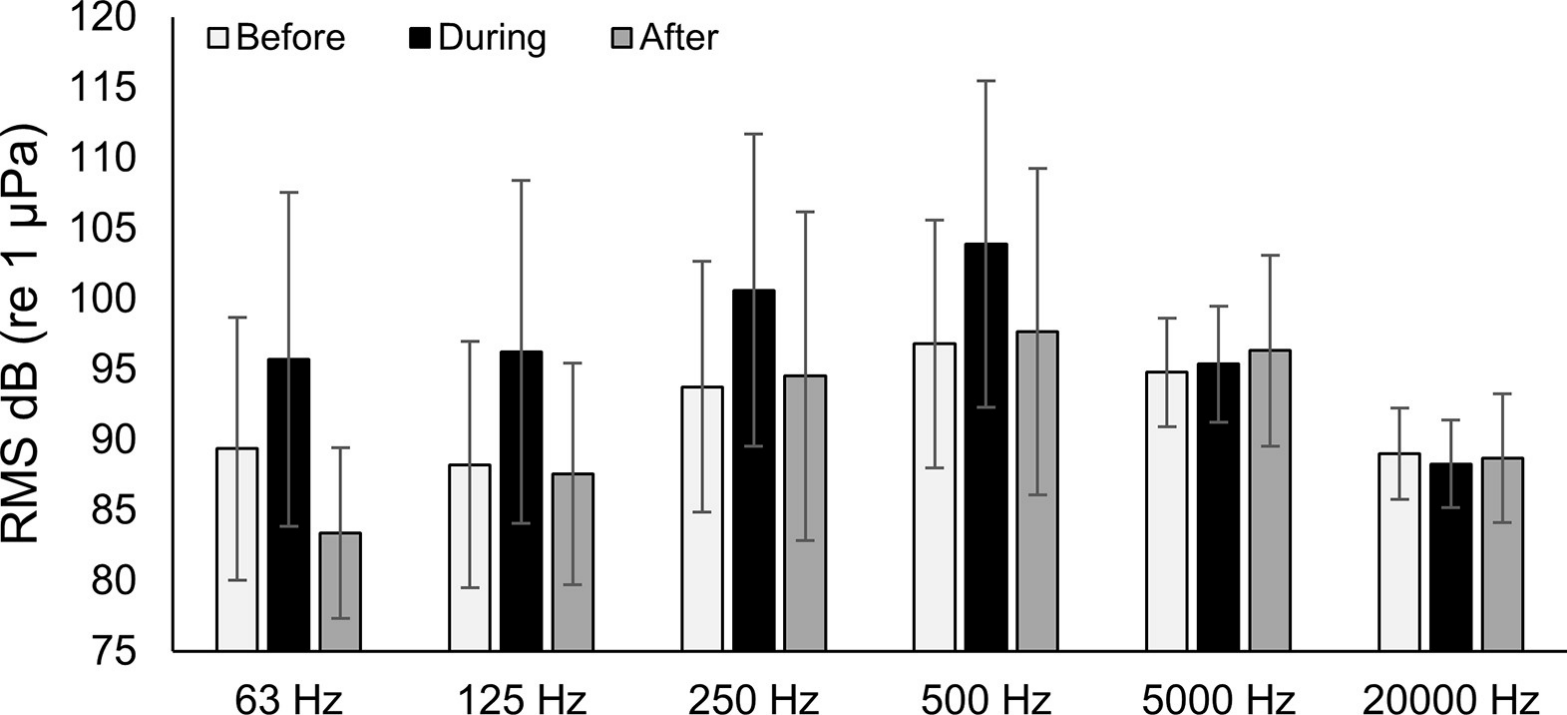
Boca 3 mean RMS sound pressure levels. Mean RMS sound pressure level at station Boca 3 (inshore) third-octave bands for before, during and after the passage of Tropical Storm Debby. Error bars ± 1 SD.

At station Boca 3 (inshore), all third-octave bands with center frequencies up to and including 500 Hz noticeably increased in amplitude during the storm (Figs 3 and 4). Mean RMS sound pressure levels during the storm were as high as 12.3 dB above mean levels before and after the storm (63 Hz center frequency). The highest mean value was 103.9 dB re 1 μPa (500 Hz center frequency during the storm), and the highest single measured RMS sound pressure level was 126.4 dB re 1 μPa (500 Hz center frequency, June 24 18:10 hrs). In the 5000 Hz and 20000 Hz bands, the maximum mean values did not occur during the storm, but instead occurred after the storm (Figs 3 and 4). These bands also showed considerably less variability in their mean sound pressure levels than was observed in lower frequency bands. Repeated measures ANOVA indicated that significant differences occurred in the RMS sound pressure levels before, during and after the passage of Tropical Storm Debby at station Boca 3 (Table 2). Post-hoc analysis indicated that sound pressure levels before and during the storm were significantly different in all bands except 5000 Hz, and that sound pressure levels during and after the storm were significantly different in all frequency bands except 20000 Hz. Sound pressure levels before and after the storm were also significantly different for all bands except 250 Hz (Table 1).

**Table 1:** Boca 3 repeated measures ANOVA and post-hoc test on sound pressure levels.

**Repeated Measures ANOVA**				
**Effect**	**DFn**	**DFd**	**F**	**p**
Time Period	2	14644	501.483	p<0.001*
Frequency	5	14644	609.135	p<0.001*
**Post-hoc Test**				
**Band (Hz)**	**Before storm–during storm**		**During storm–after storm**	**Before storm–after storm**
63	p<0.001*		p<0.001*	p<0.001*
125	p<0.001*		p<0.001*	p<0.001*
250	p<0.001*		p<0.001*	p = 0.004
500	p<0.001*		p<0.001*	p = 0.001*
5000	p = 0.007		p<0.001*	p = 0.001*
20000	p<0.001*		p = 0.211	p<0.001*

Repeated measures ANOVA and Tukey post-hoc test results for mean RMS sound pressure levels (dB re 1 μPa) before, during and after the Tropical Storm Debby at station Boca 3.

*Indicates significant results (p < 0.05).

**Fig 5:**
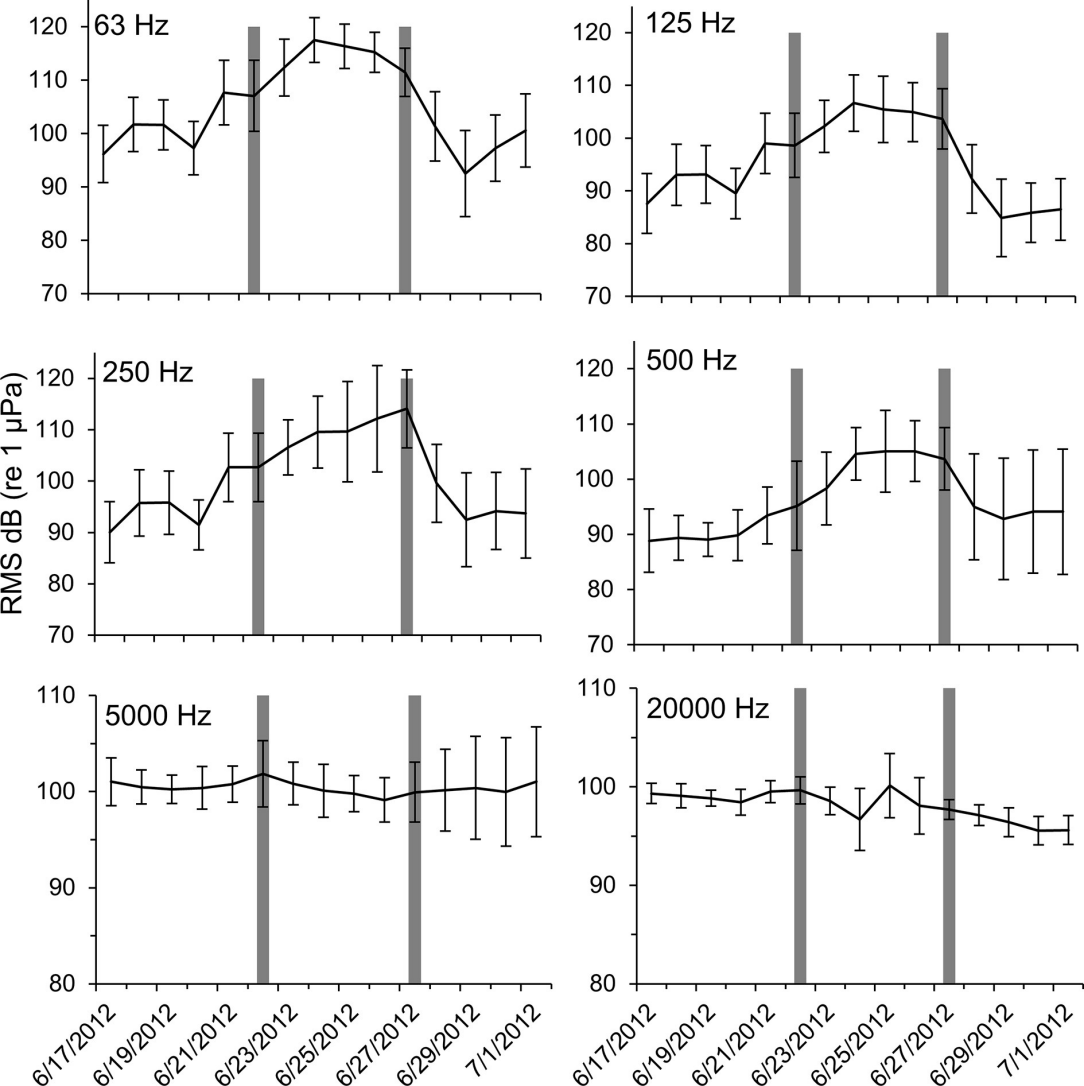
Gulf 1 sound pressure levels during Tropical Storm Debby. Daily mean RMS sound pressure levels at station Gulf 1 (offshore) for third-octave bands centered at 63 Hz, 125 Hz, 250 Hz, 500 Hz, 5000 Hz, and 20000 Hz third-octave bands. Dark bars indicate the first day of the “during storm” period, and the first day of the “after storm” period. Error bars ± 1 SD. Note that the scale of the y-axes for the top four panels are not the same as the bottom two panels.

**Fig 6:**
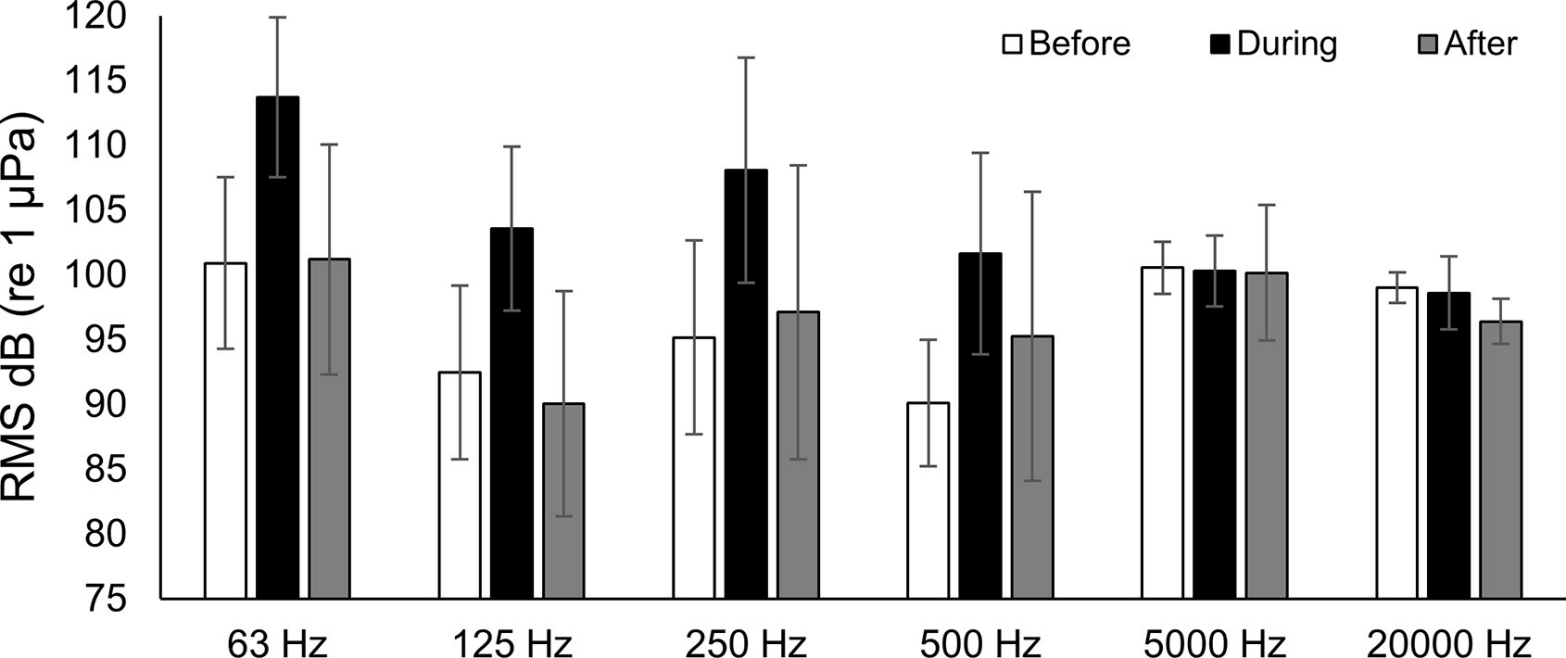
Gulf 1 mean RMS sound pressure levels. Mean RMS sound pressure level at station Gulf 1 (offshore) third-octave bands for before, during and after the passage of Tropical Storm Debby. Error bars ± 1 SD.

At station Gulf 1 (offshore), all third-octave bands with center frequencies up to and including 500 Hz also noticeably increased in amplitude during the storm (Figs 5 and 6). Mean RMS sound pressure levels during the storm were as high as 13.5 dB above levels before and after the storm (125 Hz center frequency). The highest mean value was 113.7 dB re 1 μPa (63 Hz center frequency), and the highest measured RMS sound pressure level was 127.2 dB re 1 μPa (63 Hz center frequency, June 24, 15:20 hrs). While no clear trend was seen in the 5000 Hz band, RMS sound pressure levels appeared to decrease over the study period in the 20000 Hz band (Figs 5 and 6). Results from the repeated measures ANOVA tests indicated there were significant differences in RMS sound pressure levels before, during and after the passage of Tropical Storm Debby at the offshore station Gulf 1. Post-hoc analysis indicated that sound pressure levels for before and during the storm, and for during and after the storm were significantly different in all bands except 5000 Hz (Table 2). Significant differences in sound pressure levels were also found for before and after the storm for all bands except 63 Hz and 5000 Hz (Table 2).

**Table 2:** Repeated measures ANOVA and post-hoc test for Gulf 1 sound pressure levels.

**Repeated Measures ANOVA**				
**Effect**	**DFn**	**DFd**	**F**	**p**
Time Period	2	14644	1613.177	p<0.001*
Frequency	5	14644	572.298	p<0.001*
**Post-hoc Test**				
**Band (Hz)**	**Before storm–during storm**		**During storm–after storm**	**Before storm–after storm**
63	p<0.001*		p<0.001*	p = 0.473
125	p<0.001*		p<0.001*	p<0.001*
250	p<0.001*		p<0.001*	p<0.001*
500	p<0.001*		p<0.001*	p<0.001*
5000	p<0.068		p = 0.809	p<0.080
20000	p = 0.001*		p<0.001*	p<0.001*

Repeated measures ANOVA and Tukey post-hoc test results for mean RMS sound pressure levels (dB re 1 μPa) before, during and after Tropical Storm Debby at site Gulf 1.

*Indicates significant results (p < 0.05).

### Fish calls

**Fig 7:**
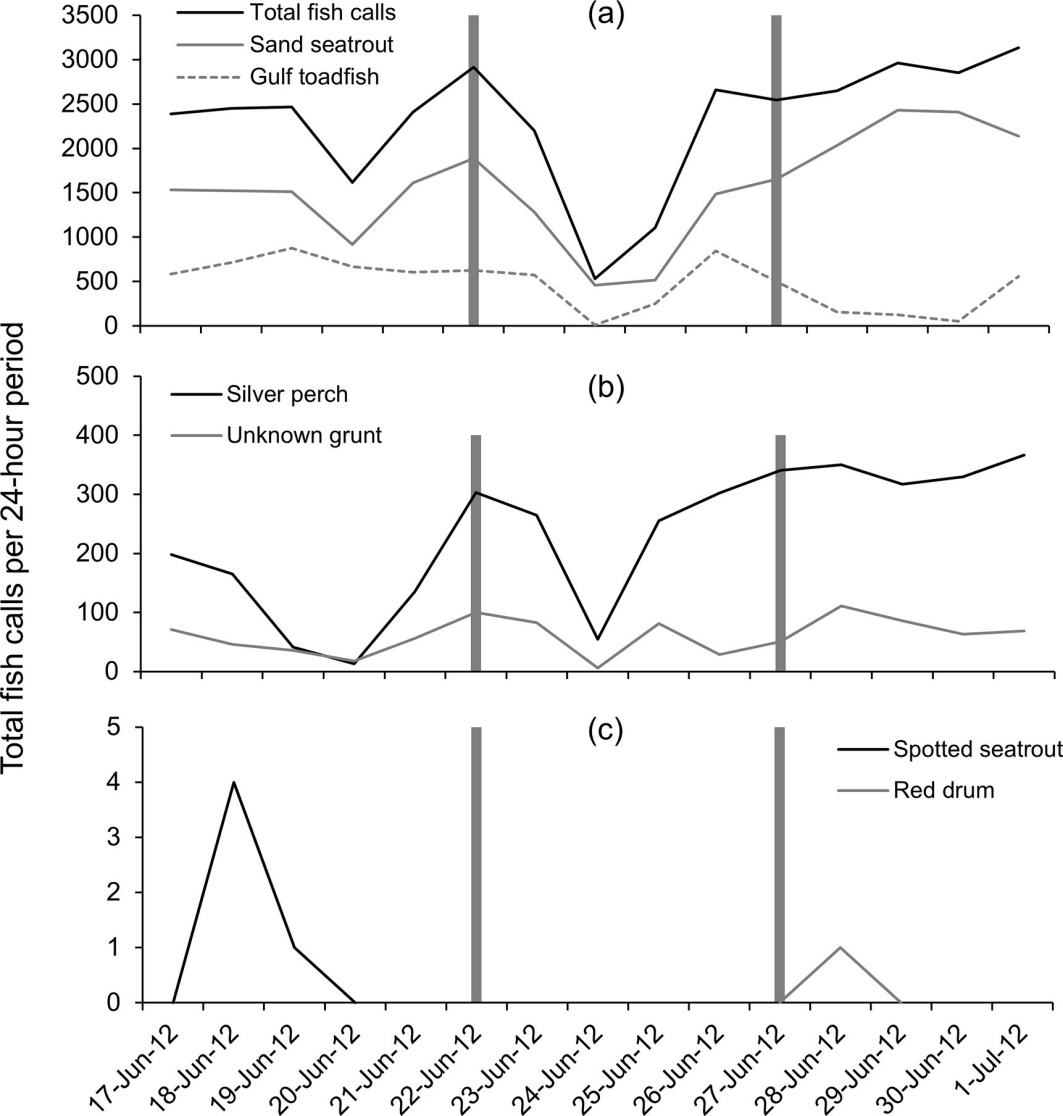
Fish detections at station Boca 3 during Tropical Storm Debby. Number of fish calls per file per 24-hour period before, during and after the passage of Tropical Storm Debby at station Boca 3 (inshore); (a) total fish calls (all species), sand seatrout calls and gulf toadfish calls, (b) silver perch, unknown grunt, (c) spotted seatrout, red drum. Dark bars indicate the first day of the “during storm” period, and the first day of the “after storm” period.

**Fig 8:**
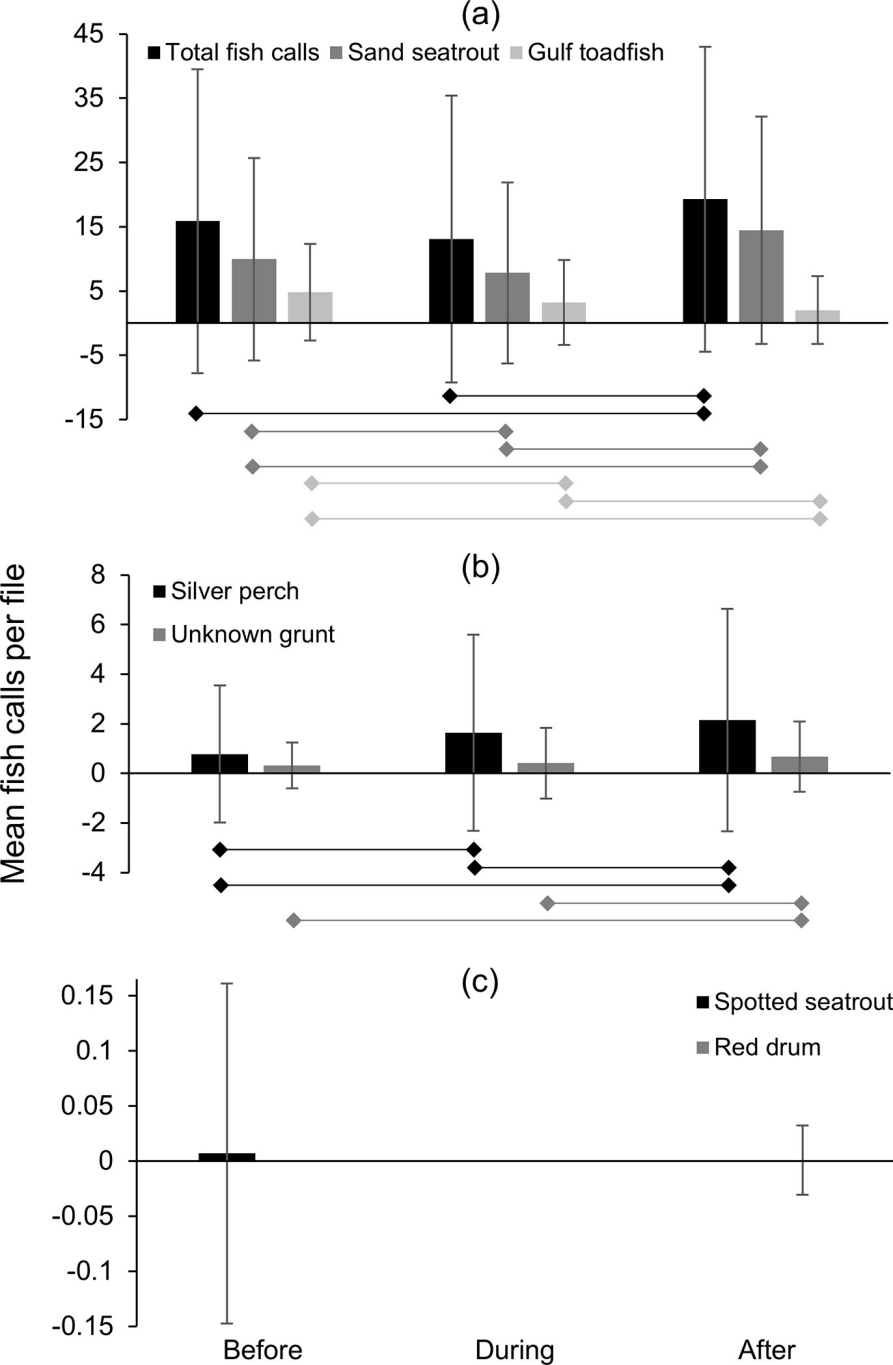
Mean fish detections per file at station Boca 3 during Tropical Storm Debby. Mean number of fish calls detected per acoustic file before, during and after the passage of Tropical Storm Debby at station Boca 3 (inshore); (a) total fish calls (all species), sand seatrout calls and gulf toadfish calls, (b) silver perch, unknown grunt, (c) spotted seatrout, red drum. Bars beneath plots indicate significant differences in post-hoc tests (p < 0.05). Note in (c) that spotted seatrout calls were only observed before the storm, and a single red drum call was observed after the storm.

Six types of fish sounds were identified to species or family level: gulf toadfish (*Opsanus beta*) silver perch (*Bairdiella chrysoura*), sand seatrout (*Cynoscion arenarius*), spotted seatrout (*Cynoscion nebulosus*), red drum (*Sciaenops ocellatus*), and grunts (*Haemulidae*). Fish calls were overall approximately twice as abundant at station Boca 3 (inshore) than at station Gulf 1 (offshore, Table 3), and at times approximately five times more abundant at station Boca 3 than at station Gulf 1 (Figs 7A and 8A). All identified calls were found at both stations Boca 3 and Gulf 1; however, spotted seatrout and red drum were only occasionally heard (Table 3).

**Table 3:** List of fish species detected at Boca 3 and Gulf 1.

Species	Boca 3	Gulf 1
Gulf toadfish (*Opsanus beta*)	7150	1635
Silver perch (*Bairdiella chrysoura*)	3437	3821
Sand seatrout (*Cynoscion arenarius*)	23399	10710
Spotted seatrout (*Cynoscion nebulosus*)	5	6
Red drum (*Sciaenops ocellatus*)	1	16
Grunt (*Haemulidae spp*.)	906	152
Total	34898	16340

List of species detected and the total number of calls at both sites during the duration of the study period.

At station Boca 3 (inshore), total fish calls (all species) per 24-hour period decreased prior to the storm but increased again before the storm affected the study area, then decreased to their lowest values during the peak of the storm (June 24, Fig 7A). Total fish calls per 24-hour period then increased to their highest values after the storm (Fig 7A). Similar patterns were also observed for sand seatrout and silver perch (Fig 7A and 7B). Gulf toadfish and grunts also had a minimum in call detections during the peak of the storm; however, this decrease was not as dramatic and unusual as seen with other species or total fish calls (Fig 7A and 7B). The calls of spotted seatrout were only detected in low numbers for two days before the storm, while a single red drum call was detected after the storm (Fig 7C). Mean fish calls per acoustic file for total fish calls (all species) and sand seatrout decreased to their lowest value during the storm and increased to their highest value after the storm (Fig 8A). However, the mean number of gulf toadfish calls decreased over the study period (Fig 8A), and the mean number of silver perch and grunt calls increased over the study period (Fig 8B). Spotted seatrout and red drum had low mean detection rates reflecting the low number of calls detected (Fig 8C)

The repeated measures ANOVA indicated that the total number of fish calls (all species combined) detected per file before, during and after Tropical Storm Debby at station Boca 3 were significantly different (Table 4). Post-hoc tests also indicated that significant differences existed between during the storm and after the storm, and before the storm and after the storm, but not before and during the storm (Table 4, Fig 8).

**Table 4:** Repeated measures ANOVA and post-hoc test on total fish detections for stations Boca 3 and Gulf 1.

**Repeated Measures ANOVA**				
**Effect**	**DFn**	**DFd**	**F**	**p**
Boca	2	2439	11.945	p<0.001*
Gulf	2	2439	59.865	p<0.001*
**Post-hoc Tests**				
**Station**	**Before storm–during storm**		**During storm–after storm**	**Before storm–after storm**
Boca 3	p<0.069		p<0.001*	p<0.001*
Gulf 1	p<0.001*		p<0.001*	p<0.001*

Repeated measures ANOVA and Tukey post-hoc test results comparing total fish detections (all species) per file before, during and after Tropical Storm Debby.

*Indicates significant results (p < 0.05).

The repeated measures ANOVA indicated significant differences between species/family-specific fish calls per file for all groups except spotted seatrout and red drum (Table 5). Post-hoc tests indicated that for gulf toadfish, silver perch and sand seatrout, there were significant differences between all pairwise comparisons (before–during the storm, during–after the storm, and before–after the storm, Table 5, Fig 8). For grunt, significant differences were found between during and after the storm, and before and after the storm, but not between before and during the storm (Table 5, Fig 8).

**Table 5:** Repeated measures ANOVA and post-hoc test on species/family-specific fish detections for station Boca 3.

**Repeated Measures ANOVA**				
**Effect**	**DFn**	**DFd**	**F**	**p**
Gulf Toadfish	2	2439	39.856	p<0.001*
Silver Perch	2	2439	25.936	p<0.001*
Spotted Seatrout	2	2439	1.781	p = 0.169
Sand Seatrout	2	2439	38.193	p<0.001*
Grunt	2	2439	17.839	p<0.001*
Red Drum	2	2439	0.711	p = 0.491
**Post-hoc Tests**				
**Species**	**Before storm–during storm**		**During storm–after storm**	**Before storm–after storm**
Gulf Toadfish	p<0.001*		p<0.001*	p<0.001*
Silver Perch	p<0.001*		p<0.008*	p<0.001*
Spotted Seatrout	p = 0.112		p = 1.000	p = 0.086
Sand Seatrout	p = 0.012*		p<0.001*	p<0.001*
Grunt	p = 0.155		p<0.001*	p<0.001*
Red Drum	p = 1.000		p = 0315	p = 0.316

Repeated measures ANOVA and Tukey post-hoc test results comparing fish species detections per file before, during and after Tropical Storm Debby at station Boca 3.

*Indicates significant results (p < 0.05).

**Fig 9:**
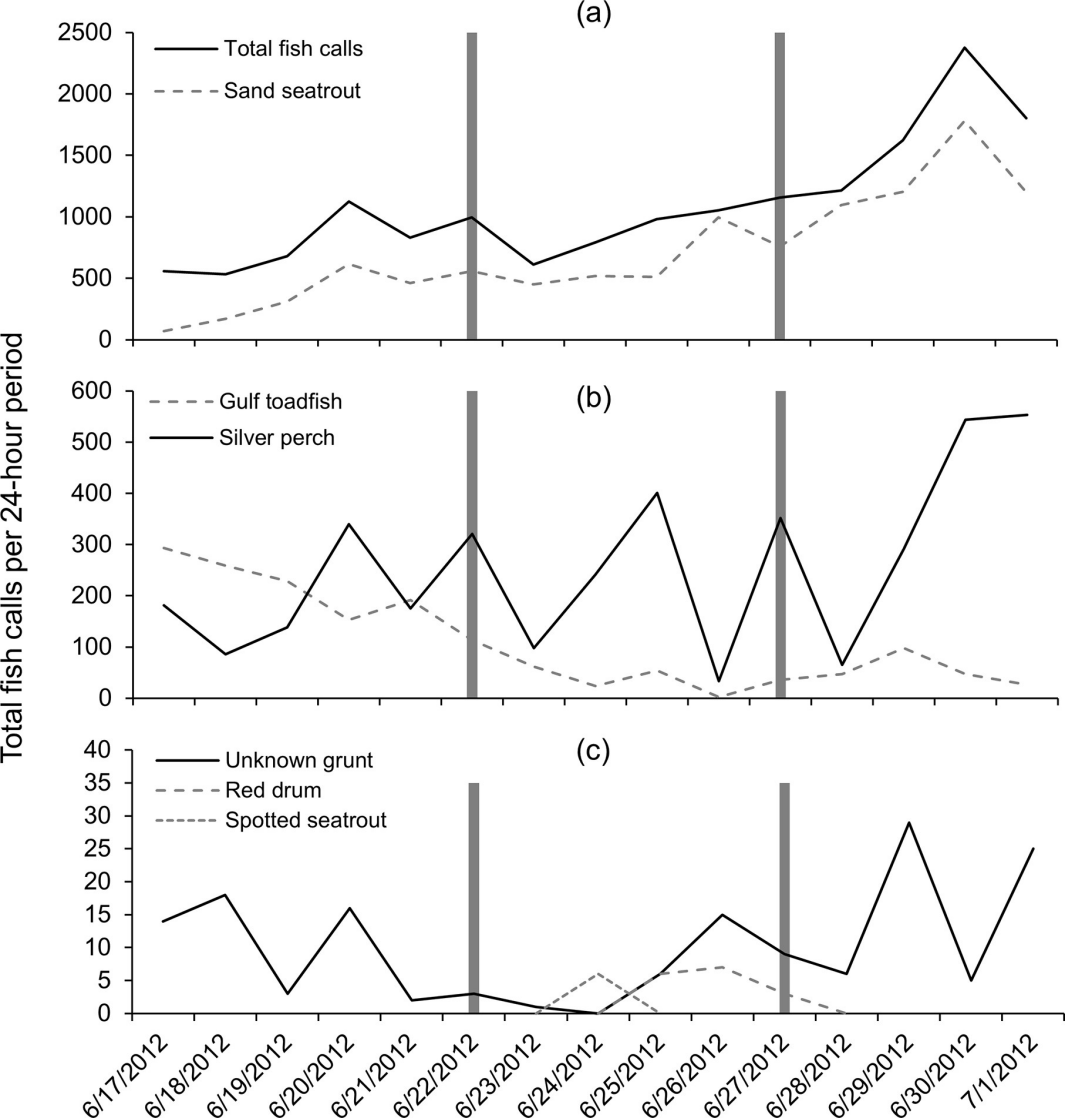
Fish detections at station Gulf 1 during Tropical Storm Debby. Number of fish calls per file per 24-hour period before, during and after the passage of Tropical Storm Debby at station Gulf 1 (offshore); (a) total fish calls (all species), sand seatrout calls (b) silver perch and gulf toadfish calls, (c) unknown grunt, spotted seatrout and red drum calls. Dark bars indicate the first day of the “during storm” period, and the first day of the “after storm” period.

At station Gulf 1 (offshore), total fish calls (all species) per 24-hour period increased prior to the storm, decreased slightly during the arrival of the storm, then increased steadily through the storm’s passage and after the storm when it peaked and declined at the end of the study period (Fig 9A). Sand seatrout calls showed a similar pattern but without a decrease during the arrival of the storm (Fig 9A). Gulf toadfish calls per 24-hour period declined to their lowest levels during storm conditions and remained relatively low after the storm (Fig 9B). Silver perch calls were highly variable throughout the study period (Fig 9B). Grunts were also highly variable but were only found at low levels during the approach of the storm (i.e., the first half of the “during” period), and red drum and spotted seatrout calls were uncommon and only found during and after the storm, or only during the storm, respectively (Fig 9C).

**Fig 10:**
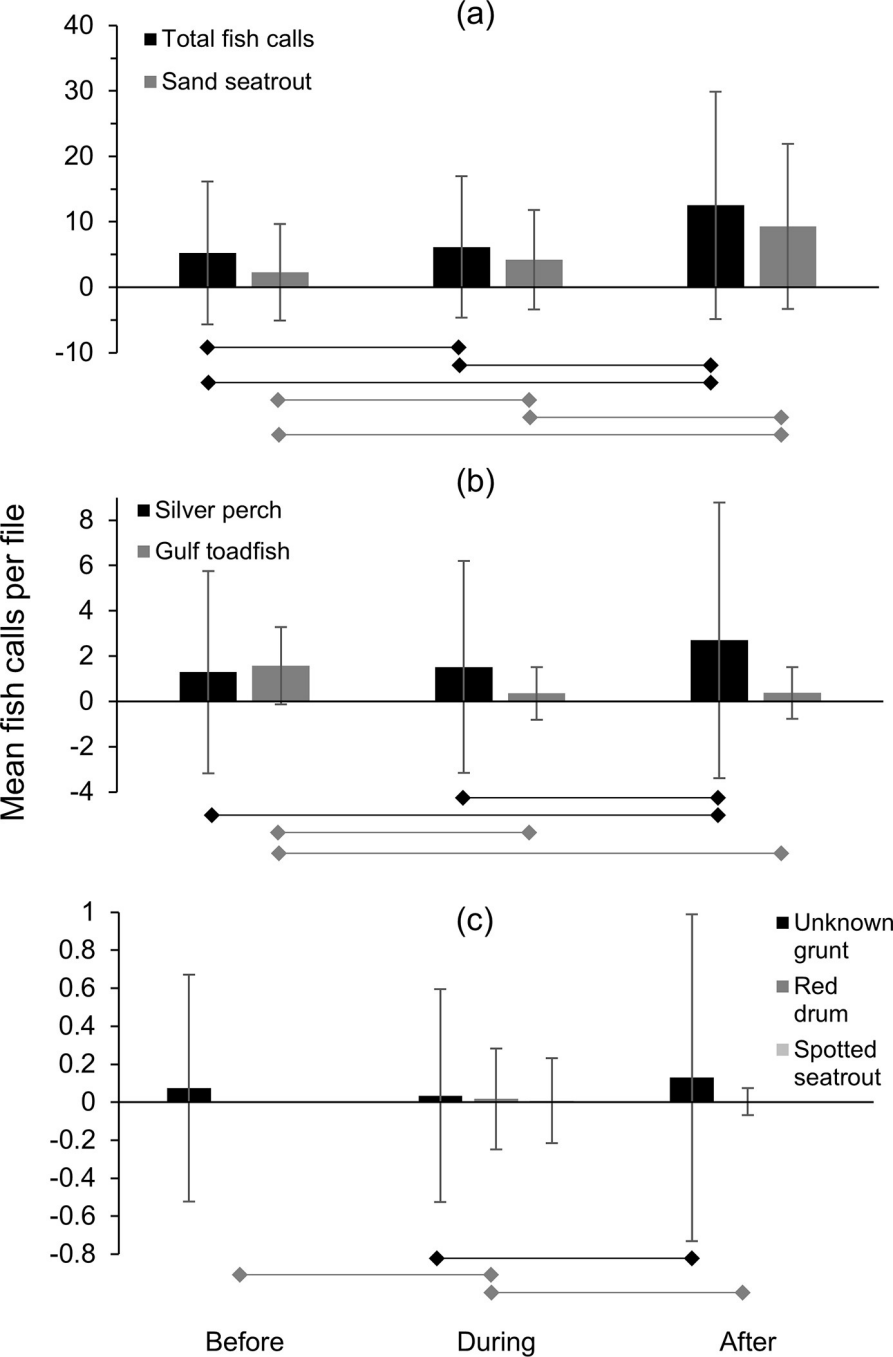
Mean fish detections per file at station Gulf 1 during Tropical Storm Debby. Mean number of fish calls detected per file per acoustic file before, during and after the passage of Tropical Storm Debby at station Gulf 1 (offshore); (a) total fish calls (all species) and sand seatrout calls (b) silver perch and gulf toadfish calls, (c) unknown grunt, red drum and spotted seatrout calls. Bars beneath plots indicate significant relationships in post-hoc tests (p < 0.05).

At station Gulf 1, mean fish calls per acoustic file for total fish calls (all species), sand seatrout and silver perch all increased throughout the study period (Fig 10A). Gulf toadfish were found at their lowest mean value during the storm, and increased slightly after the storm (Fig 10B). Grunts also were found at their lowest mean values during the storm, but increased to their highest levels after the storm. Mean fish calls per file for red drum and spotted seatrout were low (Fig 10C).

Results of the repeated measures ANOVA indicated that the number of total fish calls (all species) detected per file was significantly different before, during and after the storm at station Gulf 1 (Table 4). Post-hoc tests indicated significant differences in the number of total fish call detections between all time periods (before, during and after the storm; Table 4, Fig 10). Repeated measure ANOVAs indicated significant differences between the time periods for all species except spotted seatrout (Table 6). Post-hoc tests for species/family-specific differences found significant differences in the mean number of calls per file between all three time periods for sand seatrout (Table 6, Fig 10). For gulf toadfish, significant differences were found between before and during the storm, and between before and after the storm. Significant differences were found for silver perch between during and after the storm, and before and after the storm. For red drum, significant differences were found between before and during the storm, and between during and after the storm, while for grunt the only significant difference was for between during and after the storm (Table 6, Fig 10).

**Table 6:** Gulf 1 repeated measures ANOVA and post-hoc test on fish species detections.

**Repeated Measures ANOVA**				
**Effect**	**DFn**	**DFd**	**F**	**p**
Gulf Toadfish	2	2439	207.043	p<0.001*
Silver Perch	2	2439	18.348	p<0.001*
Spotted Seatrout	2	2439	1.196	p = 0.303
Sand Seatrout	2	2439	116.511	p<0.001*
Grunt	2	2439	3.836	p = 0.022*
Red Drum	2	2439	3.036	p = 0.048*
**Post-hoc Tests**				
**Species**	**Before storm–during storm**		**During storm–after storm**	**Before storm–after storm**
Gulf Toadfish	p<0.001*		p = 0.750	p<0.001*
Silver Perch	p = 0.402		p<0.008*	p<0.001*
Spotted Seatrout	p = 0.1940		p = 0.160	p = 1.000
Sand Seatrout	p<0.001*		p<0.001*	p<0.001*
Grunt	p = 0.291		p = 0.006*	p = 0.114
Red Drum	p = 0.025*		p = 0.041*	p = 0.687

Repeated measures ANOVA and Tukey post-hoc test results comparing fish species detections per file before, during and after Tropical Storm Debby at site Gulf.

*Indicates significant results (p < 0.05).

### Correlations

In inshore waters, several statistically significant correlations were found between environmental variables (Table 7). A negative correlation was found between water level anomaly data from the NOAA St. Petersburg weather station and in situ water temperature collected at the inshore station Boca 2 (as water level increased, water temperature decreased). In addition, a positive correlation was found between RMS sound pressure level in the 500 Hz third-octave band from station Boca 3 and the lunar cycle (as the % of the moon visible increased, sound pressure level increased). Several statistically significant correlations between fish calls recorded at station Boca 3 and environmental variables were also found (Table 7). Negative correlations were observed between total fish calls (all species), sand seatrout calls and silver perch calls and NOAA water level anomaly data (as water level anomaly increased, fish calls decreased), while positive correlations were observed between total fish calls, sand seatrout calls and sliver perch calls and bottom temperature collected at the nearby station Boca 2 (as water temperature decreased, fish calls decreased). Total fish calls, sand seatrout calls and silver perch calls were positively correlated to the lunar cycle (fish calls increased as the % of the moon visible increased), while a negative relationship was found with gulf toadfish and spotted seatrout (fish calls decreased as the % of the moon visible increased). No fish calls were correlated with ambient noise (RMS sound pressure level in the 500 Hz third-octave band).

**Table 7:** Spearman’s rho correlations for station Boca 3.

	Water level anomaly	Temperature (station Boca 2)	Lunar cycle	SPL 500 Hz third-octave band (station Boca 3)
**Total fish calls**	-0.650, p = 0.009*	0.707, p = 0.003*	0.581, p = 0.023*	-0.232, p = 0.405
**Sand seatrout**	-0.682, p = 0.005*	0.846, p<0.001*	0.574, p = 0.025*	-0.346, p = 0.206
**Gulf toadfish**	-0.025, p = 0.930	-0.150, p = 0.594	-0.695, p = 0.004*	-0.389, p = 0.152
**Silver perch**	-0.561, p = 0.030*	0.561, p = 0.030*	0.878, p<0.001*	-0.107, p = 0.704
**Unknown grunt**	-0.146, p = 0.603	0.457, p = 0.087	0.390, p = 0.151	-0.300, p = 0.277
**Spotted seatrout**	-0.278, p = 0.316	0.139, p = 0.622	-0.517, p = 0.049*	-0.290, p-0.295
**Red drum**	-0.371, p = 0.173	0.186, p = 0.508	0.248, p = 0.373	0.062, p = 0.827
**SPL 500 Hz third-octave band (station Boca 3)**	0.246, p = 0.361	-0.318, p = 0.248	0.270, p = 0.023*	-
**Lunar cycle**	-0.379, p = 0.164	0.409, p = 0.130	-	-
**Temperature (station Boca 2)**	-0.750, p = 0.001*	-	-	-

Spearman’s rho correlation results for fish calls per 24-hour time period at station Boca 3 (inshore) and environmental variables. SPL is RMS sound pressure level. All test results 2-tailed, n = 15.

*Indicates significant results (p < 0.05).

At the offshore station Gulf 1, bottom temperature was significantly and negatively correlated with both lunar cycle and the RMS sound pressure level in the 500 Hz third-octave band (as bottom temperature decreased, the % of the moon visible increased, and RMS sound pressure level increased, Table 8). The calls of grunts were negatively correlated with water level anomaly (as water level anomaly increased, fish calls decreased). Total fish calls and the calls of sand seatrout and red drum were negatively correlated with water temperature (as temperature decreased, fish calls increased), while gulf toadfish calls were positively correlated with temperature (as temperature decreased, fish calls decreased). There were positive correlations between total fish calls and sand seatrout calls and the lunar cycle (as the % of the moon visible increased, fish calls increased), however a negative correlation was found between gulf toadfish calls and the lunar cycle (as the % of the moon visible increased, fish calls decreased). Detections of gulf toadfish calls was negatively correlated with ambient noise (RMS sound pressure level in the 500 Hz third-octave band), while detections of red drum calls were positively correlated with ambient noise.

**Table 8:** Spearman’s rho correlations for station Gulf 1.

	Water level anomaly	Temperature (station Gulf 1)	Lunar cycle	SPL 500 Hz third-octave band (station Gulf 1)
**Total fish calls**	-0.429, p = 0.111	-0.518, p = 0.048*	0.765, p = 0.001*	0.225, p = 0.420
**Sand seatrout**	-0.368, p = 0.177	-0.593, p = 0.020*	0.808, p<0.001*	0.329, p = 0.232
**Gulf toadfish**	0.023, p = 0.934	0.885, p<0.001*	-0.733, p = 0.002*	-0.810, p<0.001*
**Silver perch**	-0.018, p = 0.950	-0.171, p = 0.541	0.375, p = 0.168	0.054, p = 0.850
**Unknown grunt**	-0.630, p = 0.012*	0.005, p = 0.985	0.252, p = 0.366	-0.320, p = 0.245
**Spotted seatrout**	0.433, p = 0.107	-0.247, p = 0.374	0.000, p = 1.000	0.309, p-0.262
**Red drum**	0.107, p = 0.704	-0.684, p = 0.005*	0.225, p = 0.420	0.666, p = 0.007*
**SPL 500 Hz third-octave band (station Gulf 1)**	0.389, p = 0.152	-0.857, p<0.001*	0.450, p = 0.092	-
**Lunar cycle**	-0.379, p = 0.164	-0.692, p = 0.004*	-	-
**Temperature (station Gulf 1)**	-0.007, p = 0.980	-	-	-

Spearman’s rho correlation results for fish calls per 24-hour at station Gulf 1 (offshore) and environmental variables. SPL is RMS sound pressure level. All test results 2-tailed, n = 15. Note that the test between lunar cycle and water level anomaly is the same test as in Table 7 as only one dataset for each of these variables was available.

*Indicates significant results (p < 0.05).

## Discussion

While Tropical Storm Debby did not make landfall in the direct vicinity of Tampa Bay (landfall was approximately 175 km to the north), the storm was spatially large ([10]; Fig 1) and resulted in high amounts of rain, wind, and storm surge in the area [17, 18]. We detected decreases in water temperature and increases in ambient noise at both stations (Figs 2–6), and significant variations in the call rates of some species which were correlated with storm conditions.

Water temperature near the sea floor at the inshore station Boca 2 decreased and reached minimum temperatures on June 20 (just before the storm arrived) and on June 25 and 26 (during the storm); while bottom temperature at the offshore station Gulf 1 reached a minimum value on June 27, just after the passage of the storm. After the storm passed, water temperature increased rapidly at station Boca 2, however this trend was not observed at station Gulf 1. As station Boca 2 was located in shallower, more inshore water, it is not unexpected that bottom temperatures returned to pre-storm conditions more rapidly than in deeper waters further offshore at station Gulf 1. The mechanism leading to earlier bottom cooling of inshore waters is beyond the scope of this study, but as the bathymetry of the region is very complex, it is possible that this complicated the dynamics of any upwelling of deeper water that occurred.

At both the inshore station Boca 3 and the offshore station Gulf 1, high levels of ambient noise occurred during the passage of Tropical Storm Debby that could be attributed to natural, non-biological sources (the geophony). Most of the increase in ambient noise was observed in lower frequencies (63 Hz, 125 Hz and 500 Hz third-octave bands; Figs 3 and 5), and qualitative review of these files suggested that the noise was caused by surface wind-driven waves and rain. Increases in low frequency underwater ambient noise are characteristic of large storms. Raffenberg [27], for example, reported increased ambient noise in frequencies below 10000 Hz during four hurricanes in the Bahamas, and reported that lower frequencies increased the most. Tropical storm Debby also produced heavy rain in the area, which can contribute to underwater broadband ambient noise from frequencies below 1000 Hz to above 20000 Hz [28, 29].

In addition to detecting changes in environmental conditions, acoustic monitoring is a valuable approach to detecting species and biological responses such as alterations in vocalization rates of fishes. At both station Boca 3 and Gulf 1, significant differences were found in the number of calls detected per file between the time periods before, during and after the storm for all fish species combined and for all species/group-specific calls except spotted seatrout and red drum (which were only rarely detected). However, the patterns of call production levels, and the degree of correlation between the number of calls and environmental variables, was species specific.

One pattern observed was the decrease in call numbers during the passage of the storm, followed by an increase in call numbers after the storm. This was only observed strongly with sand seatrout calls at station Boca 3 (inshore), although sand seatrout at the offshore station increased throughout the study. At Boca 3, calls were detected in high numbers before the storm, decreased to their lowest level during the storm, and increased to their highest levels after the storm. Correlations with environmental variables suggest that this was likely due to the storm conditions, as sand seatrout calls at station Boca 3 were correlated with both water level anomaly (the negative storm surge) and water temperature (which decreased during the storm). In several other cases, minimums in the mean number of fish calls were observed during the storm, but the overall pattern was only partially significant. For example, the minimum mean number of total fish calls (all species/groups) per file at station Boca 3 also occurred during the storm, but no significant difference was found between call detections before and during the storm. Similarly, gulf toadfish and grunt calls at station Gulf 1 (offshore) decreased during the storm period, however significant differences were only found between the before and during storm periods and before and after storm periods for gulf toadfish, and between the during and after storm time periods for grunt. Correlations with water level anomaly (total calls at Boca 3, grunt at Gulf 1) and water temperature (total calls at Boca 3, gulf toadfish at Gulf 1) suggest that differences in call rates were partially due to conditions associated with the storm, but other factors were likely important. As total fish calls were made up largely of sand seatrout calls, but other species also contributed and exhibited different temporal patterns, the weaker relationship with total fish calls was not surprising.

Raffenberg [27] investigated fish vocalizations (all species combined) during four hurricanes in a marine sinkhole in the Bahamas and found that vocalizations significantly decreased in the three stronger storms but not in the weaker, distant storm. However, Locascio and Mann [7] investigated fish vocalizations in the path of a category 4 hurricane (Hurricane Charlie) over a nearby area with a similar species assemblage (Charlotte Harbor, Florida). In that study, the authors found that the storm had little effect on spawning fish vocalizations (in fact, sound levels increased during and after the storm) [7]. While the study by Locascio and Mann [7] was on all fish calls combined, calls were primarily from sand seatrout. Despite the similar environments between station Boca 3 (this study) and that investigated by Locascio and Mann [7], we found that sand seatrout calls were significantly lower during Tropical Storm Debby. However, in this study and the study by Raffenburg [27], fish vocalizations were individually identified by manual inspection of spectrograms, while in Locascio and Mann [7] fish vocalizations were quantified by identifying chorusing events through spectral analysis, so the methodologies are not directly comparable.

Several types of fish calls in this study were correlated with water temperature, which at both stations appeared to decrease with the passage of the storm. At station Boca 3, total fish calls and sand seatrout calls decreased with decreasing water temperature (recorded at the nearby station Boca 2). While there has been little research done on the effect of tropical cyclones on fish vocalizations, the production rate of fish calls has been correlated with water temperature. Monczak and colleagues [30] found that negative temperature anomalies decreased the calling rates of oyster toadfish (*O*. *tau*), silver perch and spotted seatrout in the May River estuary. Mann and Grothues [31] documented declines in call rates for Atlantic croaker (*Micropogonias undualtus*) and weakfish (*Cynoscion regalis*) in response to episodic cold-water upwelling events, and a recent review by Ladich [32] indicated a general trend toward decreases in fish vocalizations in colder temperatures. This suggests that the changes in call rates observed in this study may be linked to the influx of cold water associated with Tropical Storm Debby. After the storm passed, both water temperatures and some call rates increased rapidly at station Boca 3 (the call rates possibly in conjunction with the lunar cycle). However, at station Gulf 1, temperature was not observed to increase after the storm, yet most correlations between fish calls and temperature were negative (total fish calls, sand seatrout and red drum). Therefore, fish calls increased with decreasing temperature. Only gulf toadfish had a positive correlation where the decrease in water temperature was associated with a decrease in calls. Along with a high correlation to the lunar cycle (see below), these results suggest a weaker relationship between fish call rates and the observed temperature fluctuation and a stronger relationship with lunar cycle.

As the effects of Tropical Storm Debby were severe but not extreme (e.g., wind speeds up to 15 m sec^-1^ [18]), the decreases in fish calls observed were likely due to non-lethal effects such as behavioral and distribution changes. The appearance and disappearance of species, as well as major shifts in distribution appear to be common biological reactions of fish to tropical cyclones (e.g., [3, 4, 33]). In the Gulf of Mexico, normal seasonal migrations between the Gulf of Mexico and estuaries, such as Tampa Bay, by various fish species are common (including sound producing species, e.g., sand seatrout: [34]), therefore shifts in distribution during storm events may be a common behavioral reaction.

Alternately, a reduction in the detection of fish calls during the storm could reflect a true reduction in call production by individuals. Several studies have documented reductions in sound production by fish when exposed to increased ambient noise. For example, Atlantic croaker decreased their call rates when a loud ferry passed nearby, especially during the peak calling season; [35]). Reduced fish call rates during periods of increased ambient noise have also been shown in experimental settings for gobys (*Gobiusculus flavescens* and *Pomatoschistus pictus*) which led to a decrease in spawning success [36]. However, our study found little correlation between fish calls and ambient noise, with gulf toadfish showing a strong negative correlation and red drum showing a weaker positive correlation at station Gulf 1. Higgs & Humphrey [37] also detected no correlation between ambient noise and the call rate of the goby *Neogobius melanstromus*. Although little is currently known about how free-living fish respond acoustically to increases in ambient noise levels, increases in background noise associated with tropical cyclones could potentially have profound biological consequences in terms of reductions in communication distances and other masking-related issues. For example, many fish use vocalizations in courtship and can use “eavesdropping” for activities such as foraging and avoiding predators [38–40]. If the passage of tropical storms coincides with prime spawning periods (e.g., full moon periods with certain species), the impacts of these storms could be exacerbated, especially if masking reduced the probability of finding a suitable spawning partner. The effects of ambient noise on the masking of communication signals, predator-prey relationships and reproductive efficiency of fish are considered serious and are currently areas of active investigation (e.g., see [41, 42] for reviews).

A more common temporal pattern was a consistent increase in call production over the study period. This pattern was observed strongly in silver perch at station Boca 3, and total fish calls and sand seatrout calls at station Gulf 1 (all pairwise differences significant). In these cases, weaker correlations were found between call rates and conditions associated with the storm (r < 0.60, water level anomaly and/or water temperature), but strong correlations were found with the lunar cycle (r > 0.75). This suggests that in these cases, call rates increased with the waxing moon (as the moon approached full, July 3) and that environmental conditions associated with the storm had less effect. Grunt calls at station Boca 3 and silver perch calls at station Gulf 1 appear to have been consistent before and during the storm, however, a significant increase in call rate was observed after the storm for both species. This suggests that for grunt (Boca 3) and silver perch (Gulf 1) there was an overall increase in call production with the waxing moon that was interrupted or delayed by the passage of Tropical Storm Debby. However, an increase in calls with lunar cycle could also be more sudden in these cases (i.e., call rates increase closer to the full moon), and conclusions are confounded by having no significant correlations with environmental conditions, including the lunar cycle. Gulf toadfish at station Boca 3 were the only case showing a consistent decrease in call production over the study period and were only correlated with the lunar cycle, suggesting that this species decreases its call production with the waxing moon. We expect that this was also the case with gulf toadfish at station Gulf 1, but at this location the storm conditions prematurely decreased call rates. This is supported by the strong correlation found between gulf toadfish calls and both water temperature and lunar cycle at station Gulf 1. In previous studies, the calls of spotted seatrout [43] and oyster toadfish [30, 44] have been associated with the full and/or new moons, which was attributed to the times of increased tidal flow. However, our results should be interpreted with caution. Wall and colleagues [20] found that toadfish calls (combined calls from gulf toadfish and another closely related species, the leopard toadfish, *O*. *pardus*) on the West Florida Shelf were not associated with the lunar cycle [20]. In a nearby estuary, sand seatrout were also not found to have a lunar periodicity in sound production [45]. As our study took place during a single phase of the lunar cycle, we can only suggest the relationship between fish calls and the lunar cycle as a possibility, and studies over multiple lunar cycles are needed to confirm this pattern.

Overall, the changes in fish calling rates appear to be partly due to storm conditions but are likely also the result of other factors including the lunar cycle. The call rates of sand seatrout suggest that the effects of the storm on the fish community were more severe at station Boca 3. For sand seatrout, call rates were significantly lower during the storm at station Boca 3, while at Gulf 1 calls increased throughout the study despite the storm and were strongly correlated with lunar cycle. Station Boca 3 was located close to shore and in shallow water (3 m), and therefore was likely exposed to a more significant storm surge, a greater freshwater input with the accompanying chemical and solid waste pollution associated with major storm events, and more water motion from surface waves and currents. Station Gulf 1, on the other hand, was in approximately 9 m of water and approximately 10 km from shore. Although it was more exposed to ocean swell due to its location in the Gulf of Mexico, at 9 m depth the conditions may have been less severe, which could mean the fish community may have been less impacted by the storm. Greater impacts on the fish community from tropical cyclones in shallow water environments is a pattern that has been reported previously [46]. This difference in depth may also change the acoustic field surrounding the stations, and therefore could also affect fish call detections.

Most or all species appeared to recover very quickly from this tropical storm. Rapid recovery of fish communities after tropical cyclones have also been documented in other areas (e.g., in the Indian River Lagoon, Florida, USA after Hurricanes Frances and Jeanne [47]). Increases in call detections at the end of the study period can be explained by immigration of individuals into the area, or by an increase in sound production by the individual fish already present (e.g., an increase in call rate due to the lunar cycle). Despite the apparent resistance and resilience to Tropical Storm Debby in this study, given the other evidence for effects of tropical cyclones on fish communities (e.g., [46, 48]) further research should be conducted in this area.

While acoustic masking of fish calls by the noise of the storm may contribute to the decrease in call detections in this study, we believe that this effect is low. For several species, the rate in fish call detections increased steadily throughout the study (e.g., sand seatrout at station Gulf 1) or decreased steadily through the study (gulf toadfish at station Boca 3) despite significant increases in ambient noise during the storm. If significant masking were taking place, we would not expect call rates to be higher than in a storm-free period (i.e., before or after). In addition, while the rate of fish calls were significantly correlated to other factors (e.g., water temperature), fish calls were only correlated with ambient noise for gulf toadfish at station Gulf 1. Therefore, while signal masking may be a factor in our results, we believe this bias does not completely explain the observed patterns in fish detections. Masking by anthropogenic sounds (mostly from boats) could potentially occur as well, it would occur mainly outside the period of the storm’s passage, especially before. Therefore this would bias our results to decreased call detections before and after the storm. We cannot rule out this possibility, however as the highest levels of ambient noise were ubiquitously during the storm, we believe that this effect is minimal.

## Conclusion

This study is one of only a few studies to examine the soundscape of a tropical storm. We found significantly higher ambient noise levels during Tropical Storm Debby, and noise levels increased most significantly at lower frequencies (up to the 500 Hz third-octave band). These noise levels, as high as 127.2 dB_RMS_ re 1 μPa and up to 13.5 dB above mean background noise levels, are expected to have a significant impact on marine life that uses sound for foraging, communication and predator avoidance. This study is also only the third study that we are aware of to investigate fish sound production immediately before, during, and immediately after a tropical cyclone, and the first to do so with species/group specific sounds. The use of PAM technology allowed us to observe the biological effects of a tropical storm at a far higher temporal resolution than possible using many other methodologies. In both the inshore lagoon ecosystem of Boca Ciega Bay and the deeper water Gulf of Mexico ecosystem, the detection rates for some fish calls appeared to have a biological reaction to the passage of Tropical Storm Debby in that fish sound production decreased, but as fish sound production recovered quickly this ecosystem showed both resistance and resilience to this disturbance. However, other species’ call rates did not appear to be strongly affected by the storm, and fish calls were highly correlated with lunar cycle. It is important to remember that this rapid recovery and lack of response in some species would potentially not apply to a direct impact from a major tropical cyclone. Within a 200 km radius, the Tampa Bay area had 21 tropical cyclones between 2001 and 2020, and 121 tropical cyclones (including 16 category 3–5 hurricanes, [49]). While this rate is similar to other areas experiencing tropical cyclones (e.g., Miami, USA and Nassau, Bahamas), the area has been exposed to fewer major hurricanes (category 3–5) during those times [49]. Studies investigating major hurricanes have reported fish mortality, loss of diversity and decreased sound production [27, 47]. However, recovery of the fish community after a major hurricane can be observed in days to weeks after the passage of the storm [27, 47]. Therefore, our results and the results of previous studies indicate that fish communities generally have high levels of resistance and resilience to tropical cyclones. The cumulative and synergistic effects of tropical storms also need to be considered as coastal ecosystems are also besieged by climate change, overfishing, pollution and other threats. In addition, there is compelling evidence that global climate change is increasing the frequency and intensity of tropical cyclones [11–13]. Therefore, it is increasingly important to understand how marine (and other) ecosystems respond to and recover from these storms.

## Supporting information

S1 File Raw data. The spreadsheet contains the fish vocalization raw count data per file throughout the study period.
(XLSX)
