# Supplementary material for: Tropical Storm Debby: Soundscape and fish sound production in Tampa Bay and the Gulf of Mexico
Source: PLoS One. 2021 Jul 13;16(7):e0254614. doi: 10.1371/journal.pone.0254614 (PMC8277075; doi:10.1371/journal.pone.0254614)
Supplement: Attachment — Submitted filename: FINAL_Response_Letter_Soundscape_Manuscript.docx.pdf [file pone.0254614.s002.pdf]

Duke | NICHOLAS SCHOOL OF THE ENVIRONMENT
DUKE UNIVERSITY MARINE LAB

June 1, 2021

Dear Dr. Higgs,

Thank you for the opportunity to revise our manuscript: "Tropical Storm Debby: soundscape and fish sound production in Tampa Bay and the Gulf of Mexico." We were pleased to receive the helpful feedback from the reviewers. Incorporating their constructive responses helped to significantly improve the paper. Ultimately, we have followed the reviewer's recommendation to add species-specific calls to this analysis. This has increased the number of tables and figures in the manuscript. The new results have also led to new interpretation in the discussion, and required some reorganization of discussion as well.

Reviewer 1 stated the manuscript needed to be more focused, specifically on the effects of Tropical Storm Debby on fish vocalization trends. They also (along with Reviewer 2) expressed the desire to see the fish vocalization data broken down and analyzed by species. In response to these comments, we have added the species-specific data to the manuscript, along with new analysis and figures to show the species-specific vocalization trends discovered through the study (Tables 3, 5, 6, 7 & 8 and Figures 7-10). We also restructured and heavily edited the discussion section to focus on the new species-specific vocalization data. Thank you for your recommendations.

Reviewer 2 also requested that the fish vocalization data be analyzed on a more granular level, in addition to suggesting a new way to display the data in Figure 7. Thus, we added the species-specific vocalization data to the manuscript and replaced Figure 7 with Figures 8 and 10. Furthermore, they recommended that we speak more to the disadvantages and limitations of PAM methodologies in the discussion section. We elaborated on the limitations of PAM methodologies in the discussion and the limitations of our study design as a whole. We believe these changes greatly enhance the manuscript and thank the reviewer for raising these points.

We have also amended our funding statement and competing interests statement as requested by the editor. The new statements are as follows:

Funding Statement: The work was supported by NOAA Ernest F. Hollings Undergraduate Scholarship (awarded to A.D.B. | https://www.noaa.gov/office-education/hollings-scholarship). Development and construction of the Digital SpectroGram recorders built in this study was supported by a grant (awarded to P.S.) from the National Oceanographic Partnership Program (OCE-0741705 | https://www.nopp.org). The funders had no role in study design, data collection and analysis, decision to publish, or preparation of the manuscript. The funder, Loggerhead Instruments (Sarasota, Florida, United States of America), provided support in the form of salaries for authors D.M., but did not have any additional role in the study design, data collection and analysis, decision to publish, or preparation of the manuscript.

Duke University Marine Laboratory, 135 Duke Marine Lab Road, Beaufort, NC 28516-9721, USA
t: 252.504.7501 f: 252.504.7648 www.nicholas.duke.edu/marinelab

The specific roles of these authors are articulated in the ‘author contributions’ section.”

Competing Interests Statement: M.D. is affiliated with a commercial company, Loggerhead Instruments, Sarasota, Florida, United States of America, as the company's President. This does not alter our adherence to PLOS ONE policies on sharing data and materials.

We have addressed all other major and minor concerns of both reviewers and have detailed those responses below in a point-by-point discussion and by highlighting where in the manuscript we have made suggested changes. We feel that the manuscript was improved with these changes, and we thank the reviewers for their time and insight.

We look forward to your thoughts on our revised work! Please let me know if there is anything else that you may need.

Sincerely,

Anjali D. Boyd

Corresponding Author
PhD Student
Duke University Marine Lab

We very much appreciate the time and effort that the reviewers gave to our manuscript. Below we have provided a detailed and specific response to reviewers' criticisms. Our responses are shaded blue for ease of reference.

Editor's Comments:

Comment 1. Please ensure that your manuscript meets PLOS ONE's style requirements, including those for file naming. The PLOS ONE style templates can be found at:

Reply: We have rechecked the revised manuscript's format to ensure it meets the PLOS ONE style requirement.

Comment 2. We note that one or more of the authors are employed by a commercial company: Loggerhead Instruments, Sarasota, Florida, United States of America. Please provide an amended Funding Statement declaring this commercial affiliation, as well as a statement regarding the Role of Funders in your study. If the funding organization did not play a role in the study design, data collection and analysis, decision to publish, or preparation of the manuscript and only provided financial support in the form of authors' salaries and/or research materials, please review your statements relating to the author contributions, and ensure you have specifically and accurately indicated the role(s) that these authors had in your study. You can update author roles in the Author Contributions section of the online submission form.

Please also include the following statement within your amended Funding Statement.

> "The funder provided support in the form of salaries for authors [insert relevant initials], but did not have any additional role in the study design, data collection and analysis, decision to publish, or preparation of the manuscript. The specific roles of these authors are articulated in the 'author contributions' section."

If your commercial affiliation did play a role in your study, please state and explain this role within your updated Funding Statement.

Reply: Amended funding statement:

The work was supported by NOAA Ernest F. Hollings Undergraduate Scholarship (awarded to A.D.B. | https://www.noaa.gov/office-education/hollings-scholarship). Development and construction of the Digital SpectroGram recorders built in this study was supported by a grant (awarded to P.S.) from the National Oceanographic Partnership Program (OCE-0741705 | https://www.nopp.org). The funders had no role in study design, data collection and analysis, decision to publish, or preparation of the manuscript. The funder, Loggerhead Instruments (Sarasota, Florida, United States of America), provided support in the form of salaries for authors D.M., but did not have any additional role in the study design, data collection and analysis, decision to publish, or preparation of the manuscript. The specific roles of these authors are articulated in the 'author contributions' section."

Comment 3. Please also provide an updated Competing Interests Statement declaring this commercial affiliation along with any other relevant declarations relating to employment,

consultancy, patents, products in development, or marketed products, etc. Within your Competing Interests Statement, please confirm that this commercial affiliation does not alter your adherence to all PLOS ONE policies on sharing data and materials by including the following statement: "This does not alter our adherence to PLOS ONE policies on sharing data and materials." (as detailed online in our guide for authors http://journals.plos.org/plosone/s/competing-interests) . If this adherence statement is not accurate and there are restrictions on sharing of data and/or materials, please state these. Please note that we cannot proceed with consideration of your article until this information has been declared. Please include both an updated Funding Statement and Competing Interests Statement in your cover letter. We will change the online submission form on your behalf.

Reply: Updated Competing Interests Statement:

M.D. is affiliated with a commercial company, Loggerhead Instruments, Sarasota, Florida, United States of America, as the company's President. This does not alter our adherence to PLOS ONE policies on sharing data and materials.

Comment 4. Thank you for stating the following in the Acknowledgments Section of your manuscript:

[Additionally, we would like to thank the Eckerd College Marine Science Department for their support and assistance. This recorder deployment was part of the Eckerd College Dolphin Project.]

We note that you have provided funding information that is not currently declared in your Funding Statement. However, funding information should not appear in the Acknowledgments section or other areas of your manuscript. We will only publish funding information present in the Funding Statement section of the online submission form.

Please remove any funding-related text from the manuscript and let us know how you would like to update your Funding Statement. Currently, your Funding Statement reads as follows:

[The work was supported by NOAA Ernest F. Hollings Undergraduate Scholarship (awarded to A.D.B. | https://www.noaa.gov/office-education/hollings-scholarship). Development and construction of the Digital SpectroGram recorders built in this study was supported by a grant (awarded to P.S.) from the National Oceanographic Partnership Program (OCE-0741705 | https://www.nopp.org). The funders had no role in study design, data collection and analysis, decision to publish, or preparation of the manuscript.].

Please include your amended statements within your cover letter; we will change the online submission form on your behalf.

Reply: We have updated the acknowledgements section to read as follows:

We would like to thank Kyla Foley for assistance in acoustic analysis, and all those who assisted with the deployment and retrieval of the recorders. Additionally, we would like to thank the Eckerd College Marine Science Department for their logistical support and assistance. This

recorder deployment was part of the Eckerd College Dolphin Project. Neither Eckerd College Marine Science nor the Eckerd College Dolphin Project provided funding for this project.

Comment 5. We note that Figure 1 in your submission contains map images which may be copyrighted. All PLOS content is published under the Creative Commons Attribution License (CC BY 4.0), which means that the manuscript, images, and Supporting Information files will be freely available online, and any third party is permitted to access, download, copy, distribute, and use these materials in any way, even commercially, with proper attribution. For these reasons, we cannot publish previously copyrighted maps or satellite images created using proprietary data, such as Google software (Google Maps, Street View, and Earth). For more information, see our copyright guidelines: http://journals.plos.org/plosone/s/licenses-and-copyright. We require you to either (1) present written permission from the copyright holder to publish these figures specifically under the CC BY 4.0 license, or (2) remove the figures from your submission:

Reply: Figure 1 was made from data downloaded from government agencies and made in ArcGIS Pro by one of the authors (PS). To be certain we are able to use this data we reached out to the appropriate agencies.

We have received email confirmation from the National Hurricane Center (6/2/21) and the Florida Fish and Wildlife Conservation Commission (6/3/21) that the data we use is public domain and we are free to use it in the figure. We also confirmed that the data used in the manuscript from the NOAA St. Petersburg weather and tide station are also public domain and available for use (confirmed via email 6/3/21). Please let me know if you need additional details.

Comment 6. In your Methods section, please provide additional information regarding the permits you obtained for the work. Please ensure you have included the full name of the authority that approved the field site access and, if no permits were required, a brief statement explaining why.

Reply: See line 106 to end of paragraph. We have also included a bit about the fact that recorders were not specifically deployed to monitor the storm, and edited the sentence to make it shorter and more readable.

Comment 7. Please include captions for your Supporting Information files at the end of your manuscript, and update any in-text citations to match accordingly. Please see our Supporting Information guidelines for more information:
http://journals.plos.org/plosone/s/supporting-information.

Reply: A caption for the supporting information file have been added to the end of the manuscript and in-text citations have been updated.

Reviewer 1:

Comment 1. I like the topic of the paper, and the idea of taking opportunistic data and finding something novel in the biological effects. Unfortunately, your main points and interesting take home messages felt a little lost in the main body of text. It felt like you were trying to put too many ideas into what should be a fairly simple paper (i.e. a storm event causing fish vocalizations to decrease due to masking/ silencing effects). I suggest you try to condense your writing and focus on the biophony aspect. I hope some of my specific comments help with this.

Reply: We appreciate this comment from Reviewer 1 and have gone through and revised the writing (specifically the Results and Discussion sections) to focus solely on the third-octave data and the fish volication data. We have completely rewritten the discussion to focus mostly on the new species-specific vocalization data that was added to the manuscript.

Comment 2. While reading the manuscript I also had a couple major questions: 1) How often storm events occur in the area? What are the knock-on effects of lots of storm events versus one or two each year? and 2) You mention Tropical Storm Debby was not a major tropical cyclone. Therefore, how often do different intensity storm events occur and what are your predictions for the relationship to sound? For example, the category 4 hurricane Charley did not inhibit fish production (line 78) but not reference the category for Debby thus making comparisons difficult.

Reply: Q1)This information and a better interpretation of the general effects of these storms are now presented in the conclusion, starting on line 664 Q2) Debby was categorized as a tropical storm, not a hurricane (category 1-5 possible with the latter). Therefore it was one category weaker than a category 1 hurricane. There are no category numbers associated with tropical storms.

Comment 3. Line 60: this sentence repeats itself, suggest you omit.

Reply: We have omitted the line.

Comment 4. Lines 66 – 76: this section is very repetitive, and I suggest could be condensed.

Reply: We have condensed this section.

Comment 5. Line 99: The authors make it seem like these recorders were placed in Tampa Bay to record the storm. However, I suspect it was serendipitous that the storm occurred, and they could look back and see the effects on noise in the area. I think the authors should be forthcoming about this as (at least in my opinion) this is a major advantage of PAM that you never truly know what you will record!

Reply: We have made it more clear that the recorders had already been deployed at our field sites for a different research project, when Tropical Storm Debby hit the area (Line 103).

Comment 6. Line 152: why was a level of 0.5m chosen as the pivotal value?

Reply: We have explained our reasoning for the 0.5m cut-off for the During the storm time period (Line 150).

Comment 7. Line 164: how were fish sounds identified to species? And are the sounds published? If not these should be included in the methodology as example spectrograms.

Reply: We have added additional information to the methods section to explain how the fish sounds were identified and added the database sources to the references (Line 166).

Comment 8. Figures 4 and 6: I suggest clustering the bar plot as frequencies, with before, during and after in different colours, as I think that would better show your differences.

Reply: Figures have been changed as suggested and we agree that they show the differences much more clearly.

Comment 9. It would also be worth knowing how far away each of the stations was from the 'epicentre' of the storm event as it passed and maybe making some predictions of source levels.

Reply: The methods section has been updated to include the distance to the track line of the storm (line 103). It is also mentioned in the first paragraph of the discussion.

We are not quite sure what the reviewer means by "source levels". In this case the source level would be the ambient noise at the surface, so therefore is a function of the distance to the water surface where the waves are being generated and rain is impacting the water surface. However we suspect the reviewer was referring to the ambient noise levels where the storm was at its maximum. This is largely a function of the relationships between wind speed / surface agitation and sound pressure level, and of rainfall and sound pressure level, at the source. We feel that attempting to estimate the sound pressure level of the storm at its maximum wind speed and rainfall is interesting but beyond the scope of this paper. Although we know of one study where a category 1 hurricane was detected acoustically 800 km away, we also feel that attempting to calculate sound pressure levels at the source is not feasible, as that signal on our recordings would be highly masked by the received levels of the local conditions.

Comment 10. Line 280: Again, I wonder how you distinguished these call types and if able to separate species could you not separate in Figure 7? Perhaps one species effected more than another? Why did you group all fish calls together?

Reply: We added the species-specific call data to the results section and refocused the discussion section on the new data added (Figures 7-10).

Comment 11. The description of environmental change with Debby at the start of the discussion although useful seems irrelevant if not linked to your main aim of investigating the effect of the storm on fish acoustic activity.

Reply: With the new addition of the species-specific data, we feel this section of the discussion is more relevant and have made it more clear how the environmental changes as a result of Debby may be responsible for some of the trends detected throughout the study.

Comment 12. At the moment the increase in sound levels during a storm event doesn't seem to be particularly novel, I think any acoustician would expect this in the low frequencies due to wind/rain noise. What is novel is linking it to fish sounds.

Reply: We have condensed this section of the manuscript and focused more on the fish vocalization trends discovered throughout our study.

Comment 13. Could you split fish species and make discussions about how the storm affects different species differently?

Reply: We have added species-specific data to the manuscript.

Comment 14. Could fish habituate to the sound of storm events if it happens regularly? I would expand on depth effects and how fish could move to deeper locations to seek acoustic refuge.

Reply: We expended on this topic in the discussion, starting at line 620,

Comment 15. Lines 420 – 431: you compare your study to others on storm events. However, you do not mention if similar species were present or even fish groups. Furthermore, were the storms during comparable times of the year? Besides methodological differences, these differences would also effect comparing results between studies.

Reply: We have added additional information (line 522) to compare and contrast the methodologies and results of similar studies to the methodologies and results from our study.

Comment 16. At the end of the discussion you deviate into talking about anthropogenic noise, I don't think this is a needed topic in this paper.

Reply: We have omitted this section from the manuscript.

Comment 17. Overall, the discussion is too long with too many ideas and little focus or direction. I suggest removing the information on geophony/anthrophony and really homing in on the biophony (both in the results and discussion) as this is the really interesting stuff!

Reply: We have restructured and rewritten the discussion to focus mostly on the species-specific trends we discovered throughout the study and omitted other information on the geophony, anthrophony, and how the storm affects other organisms (i.e. dolphins, shrimp, etc.)

Reviewer 2:

Comment 1. In the introduction, the authors state that PAM is ideal methodology to examine the biological responses to & recovery after storm. Their results however attract more questions than answers (did the fish stop calling, did they leave the area, did they die?). Although PAM can be a useful tool, in this case, it only gives us some limited information on what exactly is happening. It would be worth discussing this point made in the introduction back in the discussion.

Reply: We have expanded on this point significantly throughout our results and discussion section and discussed some of the limitations (i.e. masking) of PAM (see line 639).

Comment 2. It would be good to have some hypothesis in the introduction on to what the investigators are expecting as results, based on existing literature

Reply: We have added a line to the introduction section about our hypothesis at the beginning of this study to address this comment (Line 91).

Comment 3. Fish calls were counted for each 24-hour period: fish calls usually vary a lot during the day, did you try to calculate the fish calls per smaller amount of time, e.g. dawn/dusk, night/day? Do you think it could bring up other important trends?

Reply: We believe this is beyond the scope of this study and decided to omit this analysis. While we did perform the analysis we felt it did not add anything of substance to the study.

Comment 4. L 186: please explain how exactly you could determine if masking was affecting your rates, it is unclear

Reply: The discussion of why masking is thought to be a factor but a minor one has been elaborated on to hopefully be clearer (starting on line 636).

Comment 5. Fig. 7: how is it possible to get a negative mean number? I think this data should be presented as boxplots rather than histograms

Reply: We added new results to the results section and conducted new analysis, the old Fig 7 was replaced by Figures 8 and 10.

Comment 6. L 378: avoid double negative 'not unexpected'

Reply: This has been fixed.

Comment 7. L 382 – 393: could the change of water depth during the storm also have altered the ambient noise propagation?

Reply: Yes, this is now acknowledged in the discussion (starting on line 627).

Comment 8. L 196 – 502: How can you be sure that anthropogenic noise did not mask fish calls significantly during your study?

Reply: This is now discussed starting on line 647.
